# Public Health's Next Step in Advancing Equity: Re-evaluating Epistemological Assumptions to Move Social Determinants From Theory to Practice

**DOI:** 10.3389/fpubh.2020.00131

**Published:** 2020-05-07

**Authors:** Tasha L. Golden, Monica L. Wendel

**Affiliations:** ^1^Department of Health Promotion and Behavioral Sciences, School of Public Health and Information Sciences, University of Louisville, Louisville, KY, United States; ^2^International Arts + Mind Lab, Brain Science Institute, Johns Hopkins University School of Medicine, Baltimore, MD, United States

**Keywords:** health equity, health disparities, social ecological, social determinants, epistemology, innovation, biomedical, research methods

## Abstract

The field of public health has increasingly promoted a social ecological approach to health, shifting from an individual, biomedical paradigm to a recognition of social and structural determinants of health and health equity. Yet despite this shift, public health research and practice continue to privilege individual- and interpersonal-level measurements and interventions. Rather than adapting public health practice to social ecological theory, the field has layered new concepts (“root causes,” “social determinants”) onto a biomedical paradigm—attempting to answer questions presented by the social ecological schema with practices developed in response to biomedicine. This stymies health equity work before it begins—limiting the field's ability to broaden conceptions of well-being, redress histories of inequitable knowledge valuation, and advance systems-level change. To respond effectively to our knowledge of social determinants, public health must resolve the ongoing disconnect between social ecological theory and biomedically-driven practice. To that end, this article issues a clarion call to complete the shift from a biomedical to a social ecological paradigm, and provides a basis for moving theory into practice. It examines biomedicine's foundations and limitations, glosses existing critiques of the paradigm, and describes health equity challenges presented by over-reliance on conventional practices. It then offers theoretical and epistemological direction for developing innovative social ecological strategies that advance health equity.

## Introduction

**Figure 1:**
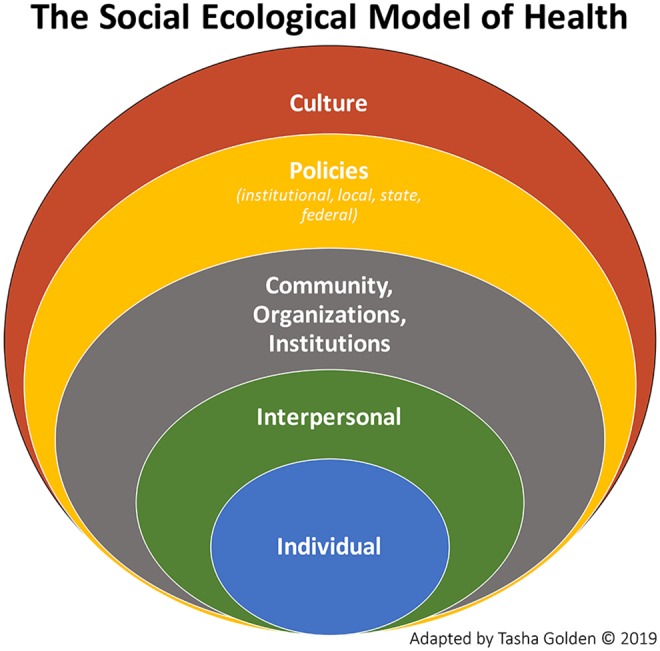
Social ecological model of health. Adapted from McLeroy et al. (6) by Tasha Golden (7).

Advancing health equity relies upon understanding the central role of social determinants of health in influencing individuals' contexts, options, and behaviors—and thus their health outcomes. Numerous studies affirm that health outcomes and disparities result not strictly or even primarily from individual behaviors or genetics, but from policies, structures, and systems that circumscribe individuals' choices, access, and knowledge (1–5). Recognizing the impacts of these factors, the field of public health has increasingly promoted a social ecological approach to understanding population health,^1^ (see Figure 1), and the language of social determinants has become a central focus in the literature (3).

Despite these changes to the field's conceptualization of health production, public health's epistemology and methodology remain rooted in the biomedical model. Research (and subsequent practice) continue to privilege individual- and interpersonal-level intervention, as evidenced by the prevailing gold standard of the randomized controlled trial, the disproportionate development and teaching of individual-level behavior theories in public health education, and the expectation among funders that measurable results at the individual level will be available within relatively short timeframes. This theory-practice dissonance in public health has generated slow progress to innovate and intervene at outer levels of the social ecological model, inabilities to access and respond to diverse knowledges, and, ultimately, failures to advance health equity [see (8)].

The work of health equity requires that public health resolve the mismatch between its historic biomedical paradigm and its evolving social ecological understanding of health. To that end, this article issues a clarion call to complete the shift from a biomedical to a social ecological paradigm. Providing a basis for moving theory into practice, this article examines biomedicine's foundations and limitations, glosses existing critiques of the paradigm, and describes health equity challenges presented by over-reliance upon—and overconfidence in—conventional methodologies. It then offers theoretical and epistemological direction for developing effective social ecological strategies that advance health equity.

## Critiquing Convention

Public health's uptake of the social ecological approach implies a critique of the driving principles of the earlier model^2^—including foundationalist ontology, positivism, reductionism, and dualism (9). However, the field has yet to make this critique explicit by consistently interrogating biomedicine's underlying principles—including to what extent those principles support and advance social ecological practice. Unfortunately, without consciously interrogating and adapting the *foundations* of biomedicine and conventional health approaches, public health cannot execute an equity-advancing response to the social ecological perspective^3^. Instead, researchers and practitioners are likely to graft new concepts (“root causes,” “social determinants”) into the existing biomedical structure—attempting to answer questions presented by the social ecological schema with practices developed in response to biomedicine.

This approach stymies health equity work before it begins, because it limits the field's ability to broaden conceptions of well-being, redress a history of inequitable valuation of knowledge and culture, or advance systemic and sociopolitical changes. The epistemological strictures of biomedicine limit public health's access to and application of diverse knowledges, resulting in obscurations and misinterpretations of behaviors and experiences that lie beyond dominant norms. These strictures also limit public health's ability to challenge the many power structures—including knowledge and evidence hierarchies—that perpetuate inequity and poor health. Indeed, throughout the twentieth century, “[a]cademic epidemiology failed to study the underlying societal factors that are causes of disturbances in health at the population level” [(11), p. 481]. In twenty-first century science, as indicated by Wemrell and colleagues' (8) review of epidemiology, “macro-level structures are still largely absent from study […while b]iomedical and lifestyle orientations thrive” (n.p.).

## Embedded Constraints

**Figure 2:**
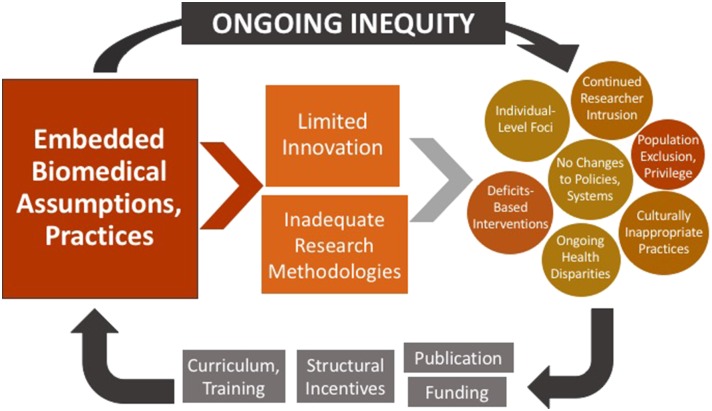
Effects of embedded biomedical practices on health equity.

Public health has faced substantial barriers to challenging or innovating standard assumptions and practices. Funding structures and requirements regularly pre-determine means by which inquiry is conducted (12) (13); similarly, the academic imperative to publish requires that researchers adhere to expectations established by editorial boards—which often hold a bias toward conventional, quantitative studies (14) (Figure 2). This reality feeds a cycle—illustrated by Figure Two—in which embedded practices perpetuate the very inequities that public health has sought to address.

As seen in this model, the entrenchment of conventional, biomedical approaches leads to limited innovation of new methods, and continued use of inadequate practices. These practices generate multiple obstacles to health equity, including continued individual-level foci, culturally inappropriate practices, deficits-based interventions, under-representation,^4^ and failures to generate systems-level change. Nevertheless, findings based on these practices continue to be published and funded—further solidifying the structural incentives related to their use, and further embedding them into public health curricula and researcher training. Health equity cannot be advanced in such an environment; like other structural determinants and health inequities, entrenched health research practices much be acknowledged, critiqued, and altered.

## Principles of Biomedicine

The biomedical model is rooted in a foundationalist ontology, “in which the world exists independently of our knowledge of it” and “unambiguous and accurate knowledge of the world” is obtainable [(16), p. 1]. This gives rise to positivist epistemology, which sees “hard, secure objective knowledge” [(17), p. 6] as both attainable and singularly valid (16). This epistemological approach drives empiricism: a focus on observation, assuming that repeated observation generates reliable, objective knowledge^5^. The biomedical model also relies on a Cartesian separation of mind and body, in which the body is a manifestation of objectively-observable causes and effects (positivism), while the mind—and “human experience” more generally—is subjective and thus unreliable, and thus minimally relevant to legitimate knowledge production or health advancement (19).

Emerging in response to the prevalence of infectious diseases as leading causes of death, the biomedical model generated profound advancements in health, including extraordinary technological and pharmacological innovations. However, its successes entrenched the model as the standard in both health care and public health practice. This remains the case despite the epidemiological transition from infectious to chronic disease. The biomedical model relies upon reductionism (20, 21), concepts of normality, and individual physiology and medicalization—to the *neglect or intentional exclusion of* social interaction, culture, systems, resources, and broad contextual factors,^6^ the impacts of which are explicitly featured by the social ecological perspective. The result of this entrenchment is a public health field stifled by increasingly inapposite expectations regarding measurement, timelines, and outcomes, and by its ongoing incapacity for engaging complexity and emergent properties.

The social ecological approach fundamentally challenges biomedicine's focus on individual-level strategies. For example, a social ecological perspective recognizes that health inequities are caused not merely by differences in behavior or genetics but by inequitable distribution of upstream drivers of health, such as education, employment, health care, and safe housing^7^. The inarguable effects of these upstream drivers challenge biomedical assumptions that health can be understood or assessed independently of social dynamics, institutional and state decisions, and historical injustices (21). Indeed, at the heart of social determinants and “health in all policies” (22) is a recognition that illness and health are largely determined by *subjective and dynamic* interpretations of human value, social norms, risks and rewards, access and opportunity, state priorities, political expediency, felt moral imperatives, etc. Public health's recognition of the impacts of such factors fundamentally problematizes our over-reliance on epistemologies and methodologies that fail to account for or interrogate them.

## Existing Critiques

Brown and Strega (10) questioned the ability of research methods grounded in positivist epistemologies to effectively address oppressive systems and social conditions, because they are themselves rooted in oppressive and inequitable structures:

Framing the discussion about what constitutes knowledge within the discourse of positivism obscures important questions about how the development of knowledge is socially constructed and controlled, how knowledge is used, and whose interests knowledge serves…[I]t fosters an illusion of neutrality or objectivity that has come to be institutionalized…as the standard by which truth claims are assessed. The racialized and gendered foundation of the Enlightenment epistemology that quantitative and qualitative approaches share is [thereby] rendered invisible, and truth claims are sequestered from questions of power, politics, and survival [(10), p. 6].

Reflecting similar concerns related to incomplete or mis-valued knowledge(s), Krieger (23) argued that “although data by themselves cannot rectify health inequities, the absence of data demonstrating [structural] harm nevertheless is itself harmful—as underscored by the time-worn adage ‘no data, no problem”' (n.p.). Despite the difficulties related to eliciting and integrating social ecological data, failures to do so perpetuate inaccurate depictions of reality that affect how and whether public health fulfills its responsibility to advance equity.

In addition, the institutionalization and standardization of such methods as randomized controlled trials has not rendered them unaffected by sociocultural assumptions regarding that which can (or should) be investigated or “reliably” known^8^. Scientific assumptions are inevitably made within or in response to biased and inequitable systems, societies, and institutions; as such, all methods require ongoing interrogation in service of increasingly inclusive, adequate sources of knowledge and knowledge production [see (8, 25–27)].

In addition to risking the perpetuation of inequity and oppression generally, positivist approaches in public health lead researchers to separate individual and social phenomena from their concomitant sociocultural meanings—including historical narratives and systemic influences, which are structural determinants of the social determinants of health. These are variously experienced, and therefore variously influential with regard to health outcomes. Thus, linear cause/effect explanations developed apart from systemic contextual understandings are likely to generate unreliable findings, which can in turn perpetuate health inequities.

Of course, as noted by Wemrell and colleagues (8), “efforts to investigate social context almost inevitably become fraught” themselves—“requiring disentanglement for the reliable measurement of individual factors” (n.p.). Yet a bias toward objectivist epistemology has perpetuated the assumption that the unreliability of decontextualized data is more scientifically acceptable than the unreliability of data resulting from investigations of social ecological contexts. This assumption is problematic, particularly when researchers from a dominant population, or who possess a privileged status, elicit or analyze data regarding populations of which they are not a part. In such situations, the ways in which participants' histories, perceptions, experiences, and meaning-making practices influence their health, health behaviors, and health disclosures may not be noticed or correctly interpreted unless researchers employ methods specifically designed to “understand others, especially those from whom [they] are … culturally remote” (28). Apart from such methods, research findings—and the interventions based upon them—may be flawed and inequitable.

Hughes and Sharrock (28) figured the significance of meaning-making and contextual understanding into their discussion of natural vs. social sciences:

The problems of the social sciences are much closer to the problem of attaining a reciprocal understanding in a conversation than they are like those of the natural scientists seeking to attain exceptionless generalizations *[sic]* for natural phenomena. That is, the methodological problems and solutions for the social sciences are of a kind involved in comprehending difficult or obscure communications and not of the sort involved in attaining valid statistical generalizations *[sic]*. [(28), p. 20].

Unlike traditional epidemiological and biomedical studies, typically understood to fall within the natural sciences, research regarding health disparities, systemic inequities, and social and structural determinants of health requires reckoning with the philosophical and methodological problems associated with the social sciences (8, 28). These include: debates over the extent to which methods designed for the natural sciences can or should be applied to the study of social phenomena (29–31); how studies will measure or address emergent properties arising out of social interaction, since these are irreducible to individual study; and how to philosophically frame—and scientifically manage—the inevitably subjective work of creating and interpreting indicators when conducting variable analyses^9^.

The “solution” to such concerns is not to discard or demonize particular methods, nor is it to simply shift methodological domination in the field to a new or currently under-used practice. Rather, given the highly contextualized nature of health represented by a social ecological approach, public health must ensure widespread training in, use of, and innovation^10^ toward *diverse knowledge-producing practices* that respond to the question of how a particular population generates and communicates knowledge.^11^ These practices must be up to the challenges of navigating subjectivity and irreducibility, interrogating science as itself a sociocultural phenomenon (24, 26, 28), and examining socio-cultural-historical narratives.

## Moving Forward

In highlighting the need to move public health from social ecological theory to practice, our goal is primarily to stimulate curiosity and innovation—directing our field's wealth of rigor and creative thought to the innovation and reformulation of epistemologies, methodologies, and practices. As a very brief beginning, we urge public health professionals to incorporate critical and hermeneutic theories into their practice(s), and to adopt elements of design thinking.

### Critical Theory

Critical theory is vital to health equity work; it calls scientists to acknowledge the extent to which the prioritization of specific methods, epistemologies, and competencies in public health maintains and exacerbates health inequities by requiring that health, health care, and health behaviors be defined, understood, and valued according to dominant ideologies and norms. Like most systems, science has been constructed via the prioritization of specific values and ways of knowing that have historically privileged specific persons and populations (10, 24, 33). As a result, scientific practice has often marginalized or erased knowledges and lived experiences that lay beyond its scope: placing them “low down on the hierarchy, beneath the required level of…scientificity” [(34), p. 82]. Health equity will not be realized unless and until public health develops methods of equitably valuing, eliciting, and responding to diverse knowledges.

Developing such methods will require increased training in (and institutional support for) transdisciplinary collaboration and design thinking (see below). It will also require surrendering the possibility of merely “import[ing] specific, fixed intervention protocols” into population health initiatives [(26), p. 1412], unreflectively “privileg[ing] quantitative knowledge over qualitative knowledge” [p. 1414], or defining success by broad scalability. Such approaches exclude or emphasize particular knowledges and populations, thus risking the distortion of findings—which can ultimately thwart scientific progress and health improvements. Foucault (34) offered a critical theoretical take on methodological hierarchies when he asked, “What types of knowledge do you want to disqualify in the very instant of your demand: ‘Is it a science?”' (p. 85).

### Hermeneutic Theory

Hermeneutic theory asserts that *meaning-making across difference* is a primary aspect of social reality; behaviors cannot be understood apart from (1) the meaning these behaviors hold for the actors themselves, and (2) the meaning those actors made of actions and circumstances that came before their own (28, 35, 36). Applied to public health, hermeneutic theory posits meaning-making as a fundamental aspect of social ecological realities and their health impacts. For example, structural and social determinants are generated, perpetuated, and shifted by dynamic attributions of meaning and value; therefore, methods failing to capture or translate meaning (and modes of meaning-making) can neither accurately assess health circumstances, nor adequately inform responsive action.

Hermeneutic theory also indicates that differences among meanings, narratives, and communicative mechanisms are to be expected—and even sought for study—rather than avoided (or manipulated into homogeneity) via the use of prescribed, ostensibly neutral communicative devices^12^. It additionally suggests that knowing how to elicit, co-create, analyze, interpret, and disseminate *multiple* modes of communication and meaning-making will generate better results than aiming for simplistic explanations.

### Design Thinking

Design thinking is an “innovative, human-centered problem-solving process” (37) that is widely used in the private sector. It challenges habitual perspectives regarding a problem, practice, or product, and supports the development and testing of new solutions. Though rarely taught to public health researchers or practitioners, integration of design thinking into public health curricula and practices could advance the field's ability to think critically and generate strategies that effectively advance health equity.

The design thinking process involves five non-linear steps: “Empathize, Define, Ideate, Prototype, and Test” (38). Echoing critical theory and the value of diverse knowledges, *Empathize* involves “set[ting] aside your own assumptions about the world” to “gain real insight into users and their needs” (38). *Define* is “an effort to explore the problem space before exploring the solution space,” and includes “end-users” (residents, clients, patients) to support optimal recognition of a problem's scope and meaning (37)^13^. The *Ideate* stage is a brainstorming process emphasizing creativity, out-of-the-box thinking, and the challenging of assumptions.

The fourth step, *Rapid Prototyping*, invites “innovators [to] iterate on theoretical and virtual prototypes until a ‘minimum awesome product' that ‘nails the pain' is created” [(39), p. 7]. Public health interventions are typically implemented at full scale after a substantive evidence base has been formed; however, the costs of this approach can preclude the exploration and piloting needed for effective progress. In contrast, “[r]apid prototyping allows for the testing of new ideas on a small-scale level and without extensive funding” (p. 7). Finally, the *Test* step in design thinking parallels evaluation research, and is meant to feed directly back into problem definition, ideation, and so on.

As noted above, advancing health equity requires public health to generate increasingly effective methodologies and interventions that go beyond individual-level strategies. These goals would benefit from design thinking's emphases on nuanced, inclusive problem-definition; unconventional ideas; rapid experimentation; and the value of “good failure” for “accelerating the learning process” [(39), p. 7]. In addition, design thinking's explicitly cyclical nature affirms that public health practices can (and should) continually be developed, honed, questioned, redefined, re-presented, and retested toward better iterations.

This is not to suggest that design thinking offers *the* solution to public health's innovation needs. It offers a beginning point: a tested model for human-centered effort that recognizes ongoing interrogation, fearless innovation, and creative (re)iteration as *non-negotiable* components of effective public health practice. As public health works to advance health and health equity, these components are critical to the development of better and more equitable research theories, methods, designs, and practices.

## Conclusion

The above opportunities describe just a few of the many ways in which public health scientists can begin rethinking epistemologies and methodologies. The field's uptake of the social ecological model, and its continued engagement with upstream drivers of health, indicates a window of opportunity to challenge the limitations of the biomedical paradigm—including the problems, research questions, and health-advancement opportunities its entrenchment has obscured.

James Baldwin wrote, “The artist cannot and must not take anything for granted, but must drive to the heart of every answer and expose the question the answer hides.” This imperative is equally applicable to scientists. While our collective assumptions, practices, and expertise in public health are typically assets, they become liabilities when they obscure the very questions necessary for transformative change (are decontextualized data reliable? Can health be assessed apart from understanding the diverse ways in which it is experienced? Are sociocultural norms and subjective experiences separable from health behaviors and outcomes?). Public health's embedded, biomedical assumptions and practices have become ready “answers” that obscure the questions and actions demanded by the social ecological model—particularly regarding structural change. In the work of advancing health equity, the questions we fail to ask—of ourselves, of our assumptions, and of our practices—are liabilities. And they are themselves determinants of health.

## Author Contributions

TG conceived of the presented ideas, wrote the body and conclusion, and conducted multiple revisions. MW contributed the introduction and revisions.

## Conflict of Interest

The authors declare that the research was conducted in the absence of any commercial or financial relationships that could be construed as a potential conflict of interest.

## Footnotes

^1^Catalyzed by the 1988 publication of McLeroy et al. (6).

^2^At the least, it implies their inadequacy.

^3^Brown and Strega (10) argue that “research cannot challenge relations of dominance and subordination“—vital to health equity work—”unless it also challenges the hegemony of current research paradigms” (p. 10).

^4^Coemans and Hannes (15) stated that “[t]raditional research methods have not always been able to fully engage with particular groups in society. This is certainly the case for vulnerable people, for instance those who have limited language abilities or those who suffered major traumas…that are difficult to verbalize” [p. 35–36].

^5^As House (18) observes, a further assumption is that reliability and objectivity together indicate truth.

^6^Such as in clinical trials.

^7^Wemrell et al. (8) provide an example of how the individual “behavior” of smoking is affected by social ecological factors: “[S]moking was typically construed as a habit of individual consumers. Other parts of the scenario…involving tobacco industry, agribusiness, or social circumstances conducive to smoking, did not provoke as much interest. As a result, smoking did indeed decrease in the western world, especially among the more privileged, while in poorer countries it increased. Despite the alleged success of epidemiology…many people still became ill due to smoking, while health disparities were exacerbated” (n.p.).

^8^Napier et al. (24) asserted that “The idea that biomedicine is able to address the ills of the world is [itself] a fundamentally cultural notion” (p. 1618), and that “biomedical knowledge is itself an artifact and outcome of cultural practices” (p. 1624).

^9^This is not meant to suggest that similar problems and considerations do not exist at all in the natural sciences.

^10^While “innovation” is often conceptualized in terms of technological advances, it is broadly needed to revamp and redesign research methods, practices, assumptions, knowledge valuations, epistemological stances, dissemination, and more.

^11^Kagawa Singer et al. (32) noted that “[i]dentifying which areas of culture may be most significant for any particular research question and population is key” (p. 243) to the development of responsive, accommodating strategies. This supports Trickett et al.'s (26) assertion that researchers would do well to move from “a ‘best practices' orientation”—which expects generalizable, ever-applicable methods—“to a ‘best processes' orientation” (p. 1412)—which is intentionally responsive to the dynamic, complex ecologies in which health research and interventions take place.

^12^Such as surveys, screenings, or even guided interviews.

^13^These steps indicate some similarities to participatory research strategies. However, Stanton (40) observed that participatory action research and community-based participatory research are more likely to invite participants into the “epistemological power structure upheld by mainstream academia” (p. 576) than to engage in critically innovative processes by which diverse knowledges are honored, utilized, and sustained. Thus even participatory action strategies require ongoing critical, epistemological interrogation. That said, such interrogation could generate effective means of revising and integrating valuable aspects of participatory strategies.
